# Calcium isotope fractionation between aqueous compounds relevant to low-temperature geochemistry, biology and medicine

**DOI:** 10.1038/srep44255

**Published:** 2017-03-09

**Authors:** Frédéric Moynier, Toshiyuki Fujii

**Affiliations:** 1 Institut de Physique du Globe de Paris, Sorbonne Paris Cité, Université Paris Diderot, CNRS, F-75005 Paris, France; 2 Institut Universitaire de France, Paris, France; 3 Division of Sustainable Energy and Environmental Engineering, Graduate School of Engineering, Osaka University, 2-1 Yamadaoka, Suita, Osaka 565-0871, Japan

## Abstract

Stable Ca isotopes are fractionated between bones, urine and blood of animals and between soils, roots and leaves of plants by >1000 ppm for the ^44^Ca/^40^Ca ratio. These isotopic variations have important implications to understand Ca transport and fluxes in living organisms; however, the mechanisms of isotopic fractionation are unclear. Here we present *ab initio* calculations for the isotopic fractionation between various aqueous species of Ca and show that this fractionation can be up to 3000 ppm. We show that the Ca isotopic fractionation between soil solutions and plant roots can be explained by the difference of isotopic fractionation between the different first shell hydration degree of Ca^2+^ and that the isotopic fractionation between roots and leaves is controlled by the precipitation of Ca-oxalates. The isotopic fractionation between blood and urine is due to the complexation of heavy Ca with citrate and oxalates in urine. Calculations are presented for additional Ca species that may be useful to interpret future Ca isotopic measurements.

Calcium is the fifth most abundant element in the Earth and meteorites^1^. It plays a central role in cosmochemical, geochemical and biological processes. It is the most refractory major element (temperature of 50% condensation of 1505 K, ref. 2) and it is therefore one of the main constituents of the first solids formed in the solar system, the so called calcium-aluminium rich inclusions. Because of its refractory behavior, calcium is generally supposed not to be lost by evaporation during planetary formation and therefore its isotopic composition has been used as a tracer of the materials that have accreted to form the Earth^2^,^3^. In surfaces environments, Ca is ubiquitous in living organisms and many minerals (e.g. calcite), and it plays a central role in the regulation of the carbon cycle and climate evolution^4^.

Calcium has 6 stable isotopes (^40^Ca, ^42^Ca, ^43^Ca, ^44^Ca^, 46^Ca, and ^48^Ca). The isotopic composition of Ca is usually reported as the δ notation as:


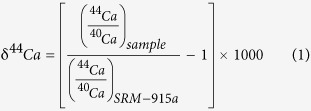


with the data reported relative to the standard NIST SRM915a^5^. SRM915a is now unavailable and has been replaced in some studies by SRM915b which is offset by ~+0.7 permil for δ^44^Ca^2^,^6^. In addition, Ca isotopic data are sometimes reported as δ^44/42^Ca, and because all the isotopic fractionation measured so far in terrestrial samples are mass dependent, δ^44/42^Ca is simply δ^44^Ca/2. For the sake of consistency, we will only refer to δ^44^Ca normalized to SRM915a in the rest of the manuscript.

Calcium isotopes have been widely used to study the paleo-variation of Ca abundance in the hydrosphere^4^,^7^,^8^,^9^ and the uptake and transport of Ca in biological materials, including plants^10^ and animals^11^. In addition, Ca isotopes have been utilized as biotracers for bone mineral balances^12^. This medical application of Ca isotopes is based on the fact that Ca isotopes are naturally fractionated between different organs and body fluids^11^,^12^,^13^,^14^. The bones are enriched in the lighter isotopes of Ca by ~1 permil compare to the blood in horses and mice^11^,^15^ and the urine is enriched in the heavy isotopes compare to the blood by ~2.4 permil as estimated from human patients^14^. Because of these isotopic variations between bones and body fluids, Ca isotopes have been used as biomarkers of change in bone mineral balances^12^. However, the origin of this isotopic fractionation between blood/urine and blood/bones is presently not understood.

Calcium isotopic variations are also a common feature in the plant kingdom. The roots are generally enriched in the lighter isotopes of Ca and the shoots are enriched in the heavier isotopes of Ca compare to the roots^10^,^16^,^17^,^18^. Again, the origin of the isotopic fractionation between the different plant components and between the roots and the source of Ca is not well understood.

It is known that large isotope fractionation during exchange reactions reflects isotopic differences between the equilibrium constants of Ca isotopologues. This comes from the fact that the partitioning isotopes of a particular element among coexisting species depend on the vibrational frequency of the bond^19^,^20^. For example, isotopic fractionation of ~1‰ was observed following cation-exchange chromatography of Ca with low (50–80%) elution yield^21^. It is therefore possible that the Ca isotopic fractionations that occur among ligands relevant to Ca species in plants, animals, water or soils may be controlled by the difference of equilibrium constants between the isotopologues. Similar mechanisms have been proposed for the origin of Zn^22^,^23^,^24^,^25^,^26^,^27^ Cu^28^,^29^, Ni^30^, Si^31^,^32^ or Fe^33^,^34^,^35^,^36^ isotope fractionations.

Here, we calculate the molecular orbitals of a large variety of Ca species to obtain the reduced partition function ratio (RPFR) of isotopologues. The *ab initio* calculations were then extended to hydrated Ca(II) complexes, chlorides, hydroxide, sulfate, carbonate, phosphates, citrates, malates, and oxalate. The choice of oxalate was motivated by evidence of isotopic fractionation of Ca between urine and blood of human patients^14^ and its importance as Ca carrier in plants^37^. Applications of our calculations to the understanding of Ca isotope variability into biological samples are briefly outlined.

## Results and Discussion

**Figure 1:**
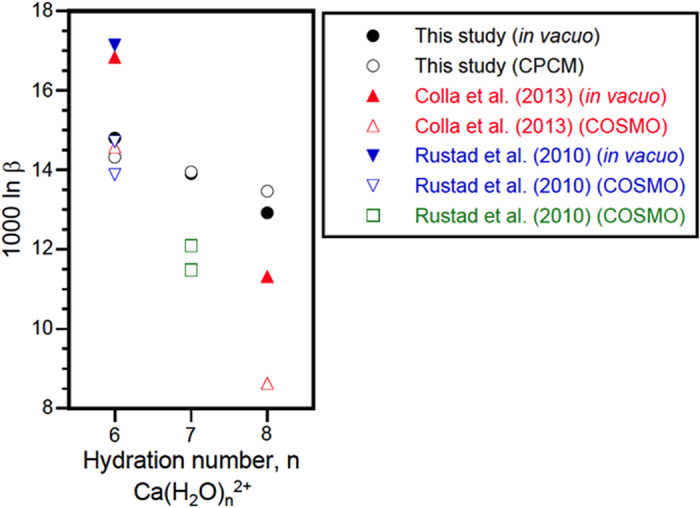
ln β of hydrated Ca^2+^ ion. The ln β values of Ca(H_2_O)_n_^2+^ (n: 6, 7, or 8) are shown together with the literature values^42^,^44^ obtained for [Ca(H_2_O)_n_](H_2_O)_m_^2+^ (n: 6 or 8, m: 12, 14, or 16) with or without the solvation model (COSMO: conductor like screening model). Solid marks (in vacuo), open marks (CPCM or COSMO)^42^.

**Figure 2:**
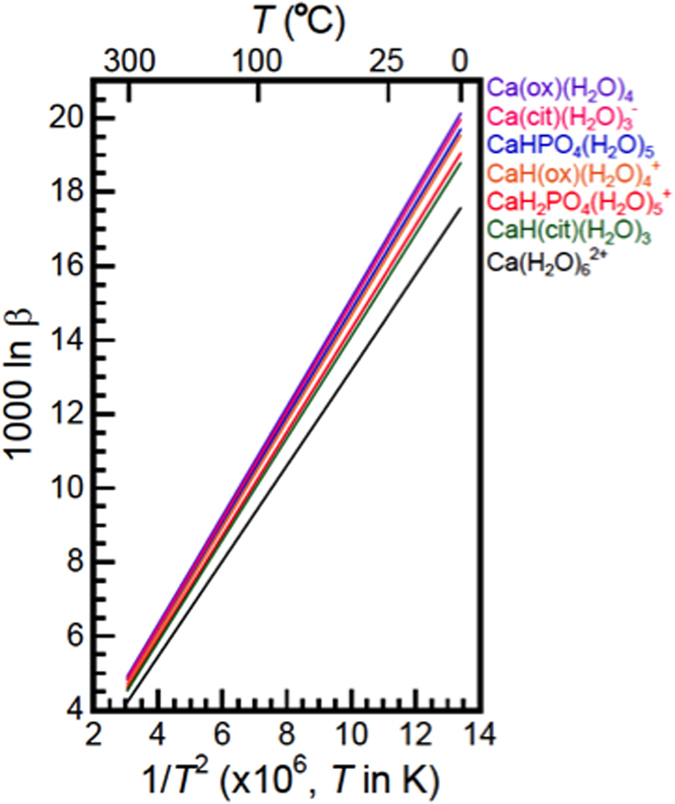
Temperature dependence of ln β. The ln β values of hydrated Ca^2+^, Ca-oxalates, Ca-citrates, and Ca-apatites are shown as linear functions of *T*^−2^.

All the data are reported in Table 1 and Figs 1 and 2 for some of the most relevant molecules discussed. The geometry of each molecules are reported in Supplementary Information.

### Hydrated Ca(II) ions

The hydrated form of the Ca(II) ion is still the subject of debate and it is usually represented on the form [Ca(H_2_O)n]^2+^ with n = 6, 7 or 8 (refs 38, 39, 40). The atomic distances between Ca and O of hydration waters in the first coordination sphere modeled as octahedral Ca(H_2_O)_6_^2+^ were analyzed to be 2.40–2.44 Å by x-ray diffraction^41^. In the present study, the Ca-O distance of Ca(H_2_O)_6_^2+^ was calculated to be 2.405 Å (in vacuo) and 2.413 Å (CPCM). This agrees with the literature data obtained both experimentally (2.41–2.45 Å)^41^ and theoretically (2.380 Å (in vacuo) and 2.4034 Å (COSMO) for Ca(H_2_O)_6_•12H_2_O)^42^.

The absolute values of ln β estimated with the large cluster model (considering first and far coordination spheres) should be more accurate than estimates based on the small cluster model (considering first coordination sphere), if the modeled complex is stereochemically correct. Introducing the solvation model (setting the dielectrics around the molecule) may improve the calculation, but its effect in the β estimation looks to be insignificant^29^. The treatment of the second coordination sphere was performed for hydrated complexes of a congener Mg^43^. Arranging water molecules in the second coordination sphere shifts 1/100 of ln β, which is much smaller than the absolute ln β value.

The computational results of hydrated Ca ion are shown in Fig. 1 together with the theoretically obtained data^42^,^44^. Our results of ln β for the sixfold coordination Ca(H_2_O)_6_^2+^ agree well with those of Colla *et al*.^42^ and Rustad *et al*.^44^. We also find that ln β decreases with increasing hydration number of the first coordination sphere from 6 to 8 (Fig. 1). On the other hand, we find that the magnitude of the isotopic fractionation between 6 and 8 is ~twice smaller than in the study of Colla *et al*.^42^. The origin of this discrepancy is unclear at the moment.

To further test the effect we have computed the ln β for seven-fold coordination of a phosphate (CaH_2_PO_4_(H_2_O)_6_^+^), a citrate (Ca(cit)(H_2_O)_4_^−^), and an oxalate (CaH(ox)(H_2_O)^5+^) (Table 1). At 310 K, their ln β values are systematically shifted by 0.7–0.9 permil compare to the six-fold coordination species similarly to what is observed for hydrated Ca molecules. Since the shift between 6- and 7-fold coordination is systematically in the same direction and magnitude, we adopt the small cluster model of six-fold coordination (*in vacuo*) for inter-molecule comparisons in the following sections.

### Application to biological activity

The application of Ca isotopes as biomarkers is based on the fact that Ca is enriched in the heavier isotopes in the order: bones, blood and urine^11^,^14^,^45^,^46^. Since >99% of the Ca budget of the human body is located in the bones, small degree of bone loss releases detectable amount of Ca in the blood stream, with Ca being isotopically lighter compared to blood background (e.g. refs 12,14,45). It is therefore possible to monitor the Ca flux out and in the bones through Ca isotopic measurements, which is relevant to bone loss conditions associated with prolonged bed rests (e.g. refs 12,14) and potentially osteoporosis. However, the origin of this isotopic fractionation has been puzzling.

In the bones, Ca is under the form of hydroxyapatite, Ca_5_(PO_4_)_3_(OH). Hydroxyapatite precipitates and mineralizes in cells called osteoblasts^47^,^48^. On the other hand, osteoclasts are the cells responsible of the bone resorption that return Ca to the blood stream^47^,^48^. The speciation of Ca in the blood is more diverse. Depending on the methods, forty five to seventy percent of the total Ca are estimated to be free (Ca^2+^) and in the biologically active form, thirty-fifty percent are bound to proteins (principally albumins and globulins) and the remaining (~10%) is complexed (e.g citrate)^49^,^50^,^51^,^52^,^53^,^54^.

For the urine, Ca is first extracted from the blood and filtrated through the glomeruli and then a major fraction (>98%) of calcium is re-adsorbed through the renal tube and the loop of Henle^54^,^55^. Part of this re-adsorption occurs through active Na^+^/Ca^2+^ exchanger selecting free Ca^2+^ and therefore leaves the excreted urine enriched in complexed Ca (Ca citrate, Ca oxalate)^54^.

From our *ab initio* calculations we can infer some of the directions of the isotopic fractionations observed between bones, blood and urine. The enrichment of ~2.4‰ of the excreted urine compare to the whole blood^14^ is consistent with the difference of speciation of Ca as the urine is enriched in citrate and oxalate Ca complexes, both species having among the highest ln β (14.685 up to 15.755 at 310 K. Table 1 and Fig. 2) compared to free Ca (l nβ up to 13.727 at 310 K) which is the dominant specie in the blood. The difference in the l nβ(citrate/oxalate) and free Ca reproduce the right order of magnitude for the difference of isotopic composition between blood and urine (~2‰).

^14^On the other hand the enrichment in the lightest isotopes of Ca in the hydroxyapatite compared to the blood is not easily explained by equilibrium between free Ca^2+^ and phosphates, which should be enriched in the heavier isotopes, as previously noted by Albarede *et al*. (ref. 56). The most straightforward explanation is that most of the (light) Ca is transferred from the free Ca specie of the blood to the osteoblasts and that reaction of apatite precipitation is quantitative and therefore not associated with further isotopic fractionation. This would leave an isotopically light bone compared to the blood.

Calcium is up taken by the plants through Ca-specific ion channels^57^. The lighter isotope enrichment of plant roots compared to the soil solutions (~1‰) has been suggested to be due to kinetic isotopic fractionation during the uptake of Ca from the soil to the roots^58^. While possible, there is however no evidence that this uptake produces kinetic isotopic fractionation. In aqueous solution, the solvation number of Ca is one of the most complex. It has a hydrated structure that have water molecules in the first coordination shell between 6 to 8 (ref. 39) while other elements like Mg has a stable 6-fold coordination^59^. Our calculations show that the l nβ of the different coordinations change by ~1‰ between 6 and 7 and by ~1‰ between 7 and 8. Calcium enters the plant cell through Ca^2+^-permeable ion channels in their plasma membranes. The ion channels are size specifics and therefore select preferentially Ca with a specific coordination. While the hydration coordination of Ca selected by ion pump is an active subject of research^60^,^61^, recent work points toward hydrated Ca^2+^ to be favored^61^. This suggests that Ca pump would preferentially select isotopically lighter Ca^2+^ and would produce isotopic fractionation between soil solutions and roots with the right order of magnitude (1–2‰), which would explain the isotopically light roots without calling for un-observed kinetic isotopic fractionations.

The enrichment in the heavier isotopes of Ca in the stems and leaves of higher plants could also be explained by a difference of coordination of Ca between the roots and the shoots. In particular, the leaves are the isotopically heavier part of a plant at >1‰ heavier than the roots^10^,^16^,^58^,^62^,^63^,^64^. Calcium-oxalates are found in the stems and leaves of all photosynthesis plants^37^. In particular, Ca-oxalates are produced in order to regulate the concentration of free Ca^2+^ in leaves following water loss by evaporation^37^,^65^. Calcium oxalates have among the highest l nβ of the Ca species (Table 1) and therefore concentrate the heavier isotopes following oxalate precipitation in the upper parts of the plants.

Isotopic fractionation of Ca at equilibrium between several species (hydrated Cu ions, hydroxides, chlorides, sulfides, sulfate, and carbonates) and organic ligands (oxalate, citrates, and malates) was demonstrated theoretically. We found that speciations of Ca can lead to large (>3‰) isotopic fractionation at 298 K. The theoretical estimation of δ^44^Ca in ligand exchange between inorganic ligands is useful to understand the natural isotopic variations in nature like the distribution of Ca within plants or animal bodies.

## Methods

Orbital geometries and vibrational frequencies of aqueous Ca(II) species were computed using the density functional theory (DFT) as implemented by the Gaussian09 code^66^. The DFT method employed here is a hybrid density functional consisting of Becke’s three-parameter non-local hybrid exchange potential (B3)^67^ with Lee-Yang-and Parr (LYP)^68^ non-local functionals. Using the 6–311 + G(d, p) basis set or higher is recommended for calculating the Mg (homologous element of Ca) complexes^43^,^44^. For Ca aquo ion, the 6–311 + G(2d, 2p) basis set was reported to be used for H and O, while the 6–311 G basis set for Ca^42^,^44^. Hence, in the present study, the 6–311 + G(d, p) basis set, which is an all-electron basis set, was chosen for H, C, N, O, F, P, S, Cl, and Ca in this study. Molecules were modeled without any forced symmetry. An “ultrafine” numerical integration grid was used and the SCF (self-consistent field) convergence criterion was set to 10^−8^. The conductor-like polarizable continuum model (CPCM) was tested to model Ca^2+^ solvation in water. All the geometry of the molecules are reported in the Supplementary Information.

The isotope enrichment factor due to intramolecular vibrations can then be evaluated from the reduced partition function ratio (*s/s*’)*f*^19^, also noted β,


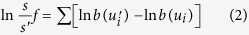


where


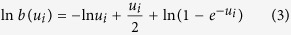


and


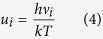


in which ν stands for vibrational frequency, *s* for the symmetry number of the Ca compound, *h* the Plank constant, *k* the Boltzmann constant, and *T* the absolute temperature. The subscript *i* denotes the *i*th mode of molecular vibration, and primed variables refer to the light isotopologue. The isotope enrichment factor due to molecular vibrations can be evaluated from the frequencies (ν) summed over all the different modes. The isotopic difference in the stability constant of chemical reactions is identical to the difference of ln β between related species. For example, a chemical exchange reaction,


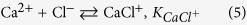


with stability constant 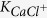, the isotope fractionation between the hydrated Ca^2+^ and CaCl^+^ is,


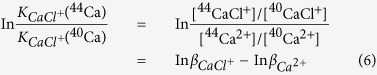


## Additional Information

**How to cite this article**: Moynier, F. and Fujii, T. Calcium isotope fractionation between aqueous compounds relevant to low-temperature geochemistry, biology and medicine. *Sci. Rep.* **7**, 44255; doi: 10.1038/srep44255 (2017).

**Publisher's note:** Springer Nature remains neutral with regard to jurisdictional claims in published maps and institutional affiliations.

## Supplementary Material

Supplementary Information

## Figures and Tables

**Table 1:** Logarithm of the reduced partition function, 1000 ln β, for the pair ^44^Ca-^40^Ca of Ca(II) complexes.

Species	Coordination number	Temperature (K)							ln β vs *T*^d^	
		273	298	310	323	373	473	573	*A*	*B*
Ca(H_2_O)_6_^2+^	6	17.483	14.802	13.727	12.687	9.611	6.049	4.148	1.2867	0.2939
Ca(H_2_O)_7_^2+^	7	16.431	13.903	12.890	11.911	9.017	5.670	3.886	1.2112	0.2565
Ca(H_2_O)_8_^2+^	8	15.273	12.918	11.975	11.064	8.372	5.261	3.606	1.1264	0.2272
CaF(H_2_O)_5_^+^^a^	6	19.444	16.506	15.324	14.180	10.779	6.813	4.685	1.4257	0.4410
CaF_2_(H_2_O)_4_^a^	6	19.003	16.139	14.987	13.871	10.553	6.681	4.598	1.3914	0.4604
CaCl(H_2_O)_5_^+^^a^	6	17.679	14.970	13.883	12.832	9.723	6.120	4.198	1.3018	0.3022
CaCl_2_(H_2_O)_4_^a^	6	16.802	14.221	13.187	12.187	9.230	5.808	3.983	1.2377	0.2760
CaSO_4_(H_2_O)_5_^a^	6	19.784	16.795	15.593	14.429	10.971	6.939	4.774	1.4498	0.4582
CaHS(H_2_O)_5_^+^^a^	6	17.181	14.544	13.487	12.465	9.442	5.942	4.076	1.2654	0.2868
Ca(HS)_2_(H_2_O)_4_^a^	6	15.108	12.785	11.854	10.955	8.296	5.219	3.579	1.1131	0.2441
CaOH(H_2_O)_5_^+^^a^	6	18.982	16.111	14.956	13.839	10.518	6.649	4.572	1.3918	0.4273
Ca(OH)_2_(H_2_O)_4_^a^	6	17.630	14.976	13.908	12.873	9.798	6.207	4.275	1.2900	0.4399
CaHCO_3_(H_2_O)_5_^+^^a^	6	19.334	16.383	15.198	14.052	10.658	6.718	4.612	1.4217	0.3637
CaHCO_3_(H_2_O)_4_^+^^b^	6	19.339	16.379	15.192	14.044	10.646	6.706	4.603	1.4230	0.3465
CaCO_3_(H_2_O)_4_^b^	6	19.070	16.182	15.022	13.898	10.565	6.682	4.598	1.3976	0.4338
CaH_2_PO_4_(H_2_O)_5_^+^^a^	6	18.953	16.057	14.894	13.771	10.442	6.581	4.518	1.3939	0.3507
CaH_2_PO_4_(H_2_O)_6_^+^^a^	7	18.075	15.307	14.197	13.124	9.947	6.265	4.299	1.3302	0.3196
CaHPO_4_(H_2_O)_5_^a^	6	19.583	16.626	15.436	14.285	10.862	6.871	4.727	1.4349	0.4564
CaH_3_SiO_4_(H_2_O)_5_^+^^a^	6	19.334	16.387	15.204	14.059	10.667	6.727	4.620	1.4210	0.3759
CaH_2_SiO_4_(H_2_O)_5_^a^	6	18.824	15.970	14.823	13.713	10.420	6.586	4.530	1.3805	0.4150
CaNO_3_(H_2_O)_5_^+^^a^	6	19.263	16.319	15.137	13.994	10.611	6.686	4.590	1.4169	0.3533
CaH(ox)(H_2_O)_4_^+^^b^	6	19.440	16.463	15.269	14.115	10.699	6.739	4.624	1.4306	0.3446
CaH(ox)(H_2_O)_5_^+^^b^	7	18.383	15.559	14.428	13.334	10.100	6.356	4.359	1.3540	0.3041
Ca(ox)(H_2_O)_4_^b^	6	20.012	16.975	15.755	14.574	11.071	6.994	4.808	1.4683	0.4302
CaH(cit)(H_2_O)_3_^c^	6	18.697	15.833	14.685	13.575	10.290	6.484	4.451	1.3755	0.3355
Ca(cit)(H_2_O)_3_^−^c	6	19.860	16.830	15.614	14.438	10.955	6.912	4.749	1.4591	0.3894
Ca(cit)(H_2_O)_4_^−^c	7	18.721	15.863	14.717	13.608	10.325	6.514	4.476	1.3755	0.3655
CaH(mal)(H_2_O)_4_^+^^b^	6	18.617	15.766	14.622	13.517	10.244	6.452	4.428	1.3701	0.3284
Ca(mal)(H_2_O)_4_^b^	6	19.139	16.217	15.044	13.910	10.552	6.655	4.571	1.4067	0.3670

^a^Counter anions are treated as monodentate ligands.

^b^Counter anions are treated as bidentate ligands.

^c^Counter anions are treated as tridentate ligands.

^d^Temperature dependence was analyzed by regression approximation, 10^3^ ln β = 10^6^ *A T*^−2^ + *B*.
