# Supplementary material for: Calcium isotope fractionation between aqueous compounds relevant to low-temperature geochemistry, biology and medicine
Source: Sci Rep. 2017 Mar 9;7:44255. doi: 10.1038/srep44255 (PMC5343585; doi:10.1038/srep44255)
Supplement: Supplementary Information [file srep44255-s1.pdf]

Supporting Information

Calcium isotope fractionation between aqueous compounds relevant to low-temperature geochemistry, biology and medicine

Frédéric Moynier[1,2] and Toshiyuki Fujii[3]

[1] Institut de Physique du Globe de Paris, Sorbonne Paris Cité, Université Paris Diderot, CNRS, F-75005 Paris, France
[2] Institut Universitaire de France, Paris, France

Table S1 Optimized structure Cartesian coordinates of Ca(II) complexes.

$Ca(H_2O)_6^{2+}$

Ca	0.000029	0.000003	-0.000042
O	2.404773	0.002355	0.000089
O	-2.404752	0.001453	0.001441
O	-0.000767	2.404899	-0.000034
O	0.003007	-2.404628	-0.000097
O	-0.000009	-0.000645	2.404701
O	0.000147	-0.000553	-2.404827
H	2.991570	0.771484	0.000250
H	2.990815	-0.767371	0.000133
H	-2.990057	0.771733	0.000951
H	-2.992325	-0.767096	0.001025
H	-0.000563	2.991297	0.769410
H	0.000012	2.991366	-0.769424
H	0.002016	-2.991022	0.769353
H	0.002305	-2.991086	-0.769498
H	0.769340	0.000422	2.991223
H	-0.769511	-0.001419	2.991022
H	0.769720	0.000306	-2.991059
H	-0.769112	-0.001110	-2.991469

$Ca(H_2O)_7^{2+}$

Ca	0.649895	0.045514	-0.229078
O	0.608890	0.310855	2.242967
O	2.927916	-0.477241	0.547770
O	1.457880	-1.831082	-1.604427
O	1.788190	2.149608	-0.598909
O	-1.008080	-1.654947	0.239641
O	-0.199569	0.465579	-2.528027
O	-1.262551	1.531461	0.142468
H	1.398620	3.025654	-0.717833

H	2.743656	2.288593	-0.561680
H	0.266120	-0.343236	2.866846
H	0.896074	1.058327	2.784293
H	-1.894710	-1.538831	0.605371
H	-0.939848	-2.589688	0.004593
H	-0.973870	0.094587	-2.971912
H	0.195294	1.076332	-3.164905
H	3.180543	-0.454818	1.479840
H	3.713586	-0.770767	0.068683
H	2.021158	-2.585001	-1.386971
H	1.270745	-1.905583	-2.549146
H	-1.584118	1.865449	0.989692
H	-1.848986	1.904189	-0.528287

$Ca(H_2O)_8^{2+}$

Ca	0.000214	0.000126	-0.000080
O	0.002844	0.111151	2.495376
O	2.376472	0.062501	0.769891
O	1.133055	-1.080741	-1.946542
O	0.352223	-2.410715	0.553609
O	1.099253	1.989249	-1.038251
O	-1.453787	0.309492	-2.007668
O	-2.228776	-1.015146	0.493281
O	-1.280075	2.034756	0.681198
H	-0.680932	-0.104611	3.141659
H	0.805024	0.290412	3.001931
H	0.642320	-3.054889	-0.104527
H	0.359353	-2.871080	1.402029
H	-2.308864	-0.130312	-2.095238
H	-1.384643	0.914910	-2.756323
H	0.862133	2.893386	-0.796465
H	1.704735	2.060902	-1.786626
H	-1.290654	2.360213	1.590106
H	-1.919713	2.570782	0.196028
H	2.938581	-0.623988	1.150141
H	2.950685	0.821708	0.608227
H	0.691375	-1.211846	-2.795018
H	2.054115	-1.336808	-2.080295
H	-3.071045	-0.609822	0.734292
H	-2.350372	-1.967870	0.592245

$CaF(H_2O)_5^{+}$

Ca	-0.365314	-0.002777	0.001721
F	1.761130	-0.000696	0.000028
O	-0.726515	-1.796795	1.588379
O	0.558159	1.954699	1.076480
O	-0.722998	1.795047	-1.582844

O	0.558409	-1.957040	-1.075983
O	-2.793341	0.003305	-0.006336
H	-0.239447	2.571400	-1.268238
H	-0.805229	1.888131	-2.538913
H	-0.819848	-1.888273	2.543561
H	-0.242892	-2.575609	1.280241
H	1.439291	-1.551192	-0.914232
H	0.623012	-2.583180	-1.803718
H	-3.375826	-0.652813	0.394786
H	-3.363786	0.666028	-0.413893
H	1.438608	1.548368	0.912854
H	0.624153	2.578652	1.805983

$CaF_2(H_2O)_4$

Ca	-0.000588	-0.005652	-0.015192
F	2.221888	-0.017629	0.007479
F	-2.222385	0.029322	0.006380
O	1.013425	1.705117	1.381505
O	1.023012	-1.720545	-1.393029
O	-1.024204	-1.382063	1.703240
O	-1.013959	1.404427	-1.714481
H	1.863644	-1.298238	-1.094128
H	1.120486	-2.026696	-2.297355
H	-1.118841	-2.291183	1.995732
H	-1.865769	-1.080233	1.285819
H	-1.080014	2.322783	-1.985960
H	-1.854292	1.130250	-1.275692
H	1.083148	1.980027	2.298629
H	1.854995	1.271279	1.104190

$CaCl(H_2O)_5^+$

Ca	-0.347815	0.022664	-0.140446
Cl	2.211553	0.358962	-0.480072
O	-0.597711	-1.425463	1.787041
O	0.306993	1.384223	1.767740
O	-0.304321	1.733820	-1.807719
O	0.200128	-1.853785	-1.511507
O	-2.766661	-0.063525	-0.010843
H	-0.769415	2.342265	-2.391616
H	0.649750	1.851737	-1.951730
H	-0.229094	-1.032369	2.589395
H	-0.469317	-2.378554	1.864068
H	1.156098	-1.763016	-1.657557
H	-0.120519	-2.566501	-2.074564
H	-3.191479	-0.541010	0.712890
H	-3.478954	0.299587	-0.550659
H	1.252500	1.484417	1.554806

H	0.045827	2.143809	2.300648

$CaCl_2(H_2O)_4$

Ca	0.000099	0.005006	0.002434
Cl	2.688271	-0.004834	0.003557
Cl	-2.688320	0.019080	-0.005349
O	0.684653	1.775524	1.495140
O	0.675096	-1.781008	-1.478608
O	-0.692890	-1.490915	1.768568
O	-0.667128	1.496578	-1.776189
H	1.637902	-1.639090	-1.450988
H	0.435797	-2.113899	-2.348413
H	-0.458521	-2.367465	2.086993
H	-1.654601	-1.457941	1.620534
H	-0.420960	2.364497	-2.108931
H	-1.630174	1.475736	-1.635143
H	0.444429	2.109918	2.364097
H	1.646890	1.629779	1.469436

$CaSO_4(H_2O)_5$

Ca	0.178189	0.221523	-0.340353
S	0.337829	-3.157730	-0.378009
O	-0.396678	0.649855	2.144098
O	2.578184	0.137288	0.114872
O	-0.045686	-0.440304	-2.365163
O	-0.360235	2.538089	0.172135
O	0.941503	-1.879513	0.223778
O	-1.115683	-3.163788	-0.016478
O	0.451872	-2.937357	-1.953315
O	1.072993	-4.360280	-0.049255
O	-1.952695	-0.784426	0.488714
H	-1.152932	0.043268	2.021655
H	0.137397	0.293782	2.862275
H	0.221494	-1.955540	-2.200598
H	-0.270493	-0.273090	-3.280749
H	-1.722255	-1.764560	0.331818
H	-2.834224	-0.657084	0.124510
H	2.495692	-0.838382	0.168440
H	3.402893	0.339038	-0.335598
H	-0.508811	3.428120	-0.158752
H	-0.499655	2.534008	1.130573

$CaHS(H_2O)_5^+$

Ca	-0.355314	-0.030718	0.077024
S	2.385303	-0.204728	-0.160047
O	-0.443671	1.833113	-1.488807
O	0.036590	-1.600792	-1.655127

O	-0.908808	-1.808060	1.625728
O	0.520237	1.540185	1.624744
O	-2.736599	0.425042	0.032939
H	-0.797410	-2.736476	1.384729
H	-0.876798	-1.778108	2.589839
H	-0.319019	1.749595	-2.442429
H	-0.011357	2.659893	-1.238917
H	1.457236	1.314444	1.414773
H	0.496254	2.186941	2.337980
H	-3.113563	1.163783	-0.460677
H	-3.475945	-0.034455	0.448454
H	1.017423	-1.519866	-1.586055
H	-0.188007	-2.172240	-2.397144
H	2.719772	-1.172609	0.717845

$Ca(HS)_2(H_2O)_4$

Ca	0.000665	-0.000953	-0.000963
S	2.304943	0.252252	1.615778
S	-2.307342	-0.248258	-1.613567
O	-1.201511	-1.994045	0.687525
O	-1.705901	1.527957	0.808873
O	1.709794	-1.530802	-0.804445
O	1.201051	1.991528	-0.692174
H	2.332138	-1.368780	-0.062689
H	2.211938	-1.791511	-1.581958
H	-1.774899	-2.061298	-0.101438
H	-0.904529	-2.878929	0.920521
H	-2.203623	1.789524	1.588947
H	-2.331754	1.369621	0.069439
H	-1.942983	-0.041160	-2.892246
H	0.906177	2.875369	-0.931601
H	1.772376	2.062589	0.097870
H	1.933135	0.046604	2.892577

$CaOH(H_2O)_5^+$

Ca	-0.157361	-0.167169	0.031576
O	-1.480015	1.743487	-0.609021
O	-1.313507	-1.808655	-1.292283
O	1.642390	-0.207910	-1.210228
O	1.140092	1.950856	0.079941
O	-1.568601	-0.206607	2.009392
O	1.523115	-1.506210	1.033023
H	1.684506	1.373515	-0.533006
H	1.732934	2.476239	0.626333
H	-2.196687	2.032454	-1.183422
H	-0.796976	2.434378	-0.606815
H	-0.814652	-2.188232	-2.026868

H	-2.148470	-2.288432	-1.249628
H	-2.230908	0.467419	2.205157
H	-1.498054	-0.755362	2.799670
H	2.046347	-2.017763	1.655528
H	2.061857	-1.278567	0.235361
H	2.101832	-0.326991	-2.042619

$Ca(OH)_2(H_2O)_4$

Ca	-0.001170	0.001950	-0.000272
O	-1.406314	-1.896283	0.650945
O	-1.878354	1.429183	0.669812
O	1.895998	0.257864	1.242723
O	1.874788	-1.429700	-0.668120
O	-1.898624	-0.257143	-1.241653
O	1.410727	1.894558	-0.652506
H	2.277082	-0.932501	0.103845
H	2.502353	-1.425320	-1.395257
H	-1.896310	-1.589411	-0.164162
H	-1.253830	-2.842434	0.586046
H	-2.505878	1.427588	1.396953
H	-2.281278	0.931527	-0.101556
H	-2.277106	-0.260985	-2.121284
H	1.265362	2.842074	-0.591621
H	1.898823	1.587146	0.163743
H	2.273405	0.261596	2.122814

$CaHCO_3(H_2O)_5^+$

Ca	0.302478	-0.127784	0.057505
O	0.026307	-0.038438	2.433888
O	2.721568	0.033251	0.193702
O	0.740862	-0.047459	-2.322556
O	-0.412023	2.058875	-0.608988
O	-0.229509	-2.452108	-0.092979
O	-1.875228	-0.415036	0.416301
O	-4.135543	-0.449620	0.411199
O	-2.987899	1.365900	-0.304832
C	-3.058092	0.050853	0.219427
H	-1.179554	-2.363078	0.103839
H	0.000054	-3.386487	-0.094333
H	-1.399122	2.027740	-0.541519
H	-0.142031	2.977880	-0.513181
H	0.745682	-0.736455	-2.997591
H	0.401708	0.753858	-2.742655
H	3.268857	0.140685	-0.594240
H	3.331010	-0.014002	0.940071
H	0.374631	0.071720	3.324521
H	-0.926307	-0.206516	2.506314

H	-3.894725	1.676656	-0.432534

$CaHCO_3(H_2O)_4^+$

Ca	0.085895	0.216320	0.045724
O	0.366528	0.623802	2.394294
O	2.362181	-0.583766	0.064837
O	0.687030	1.426974	-1.937567
O	-1.918309	1.546020	0.147288
O	-1.818069	-1.114576	-0.339370
O	-1.611789	-3.330221	-0.721300
O	0.181502	-2.069066	-0.352707
C	-1.073282	-2.139314	-0.464380
H	1.478853	1.268181	-2.466037
H	0.116294	1.997019	-2.467100
H	-2.370745	2.387250	0.267915
H	-2.586971	0.863989	-0.027015
H	1.077788	0.263161	2.937875
H	-0.287710	0.983274	3.005673
H	3.310624	-0.448411	0.159003
H	2.206127	-1.526087	-0.114303
H	-2.571872	-3.226644	-0.779021

$CaCO_3(H_2O)_4$

Ca	-0.153228	-0.361583	0.194359
O	0.204808	0.540156	2.413118
O	1.431536	0.009468	-1.551442
O	-0.454248	-2.692512	0.602768
O	-2.278850	-1.151001	-0.278204
O	-3.009293	-1.075100	-2.429862
O	-1.090243	-0.045856	-1.800965
O	-2.197028	0.833550	1.312497
C	-2.185141	-0.768545	-1.594690
H	2.236941	0.122831	-2.060244
H	0.644368	0.018109	-2.152418
H	-0.727515	0.809463	2.526750
H	0.614521	0.452640	3.277689
H	-2.542503	0.069194	0.735821
H	-2.700310	1.617090	1.070806
H	-0.240645	-3.623751	0.510080
H	-1.367161	-2.513244	0.235907

$CaH_2PO_4(H_2O)_5^+$

Ca	0.190813	0.136969	-0.016955
P	0.174354	-3.218341	-0.516120
O	-0.315835	0.590066	2.312375
O	2.560600	0.118387	0.230510
O	-0.057239	-0.269087	-2.362126

O	-0.317600	2.507512	-0.087280
O	0.955304	-1.978326	-0.088810
O	-1.219496	-3.343252	0.038447
O	0.176217	-3.065080	-2.154425
O	1.029821	-4.559679	-0.297990
O	-1.922274	-0.894362	0.581467
H	-1.080868	0.019017	2.480219
H	0.177591	0.665047	3.136930
H	0.067760	-1.221850	-2.555679
H	-0.302604	0.160646	-3.187286
H	-1.803226	-1.889558	0.439060
H	-2.845834	-0.697818	0.392030
H	2.696014	-0.840596	0.140924
H	3.418940	0.550480	0.273101
H	-0.289488	3.206775	-0.750640
H	-0.606034	2.921045	0.736205
H	0.627391	-5.164809	0.337525
H	-0.199073	-3.817091	-2.630527

$CaH_2PO_4(H_2O)_6^+$

Ca	-0.068126	-0.217964	0.141225
P	0.313021	-0.307442	3.716616
O	1.538684	-2.233217	0.392554
O	-0.651954	-2.138000	-1.198675
O	0.495374	2.008235	1.056731
O	-1.689760	1.061881	-1.163396
O	-1.340030	-0.174980	3.790000
O	0.775176	1.286623	3.673547
O	0.870296	-1.004418	4.889137
O	1.658616	0.813775	-1.214051
O	-2.294739	-0.332644	1.218055
O	0.521322	-0.740047	2.265774
H	-2.535743	1.157291	-0.707630
H	-1.746495	1.546347	-1.993804
H	1.144771	1.588872	4.512775
H	-1.681119	-0.237749	4.691321
H	2.294972	0.595656	-1.903092
H	1.975032	1.607677	-0.762641
H	2.441406	-2.518813	0.218090
H	1.466720	-2.067397	1.355179
H	-1.262675	-2.490810	-1.853189
H	-0.044557	-2.843553	-0.931218
H	0.033854	2.844877	0.935801
H	0.708073	1.943278	2.015044
H	-2.952201	-1.026317	1.096127
H	-2.150708	-0.268794	2.189032

$CaHPO_4(H_2O)_5$

Ca	0.200258	0.214528	-0.346793
P	0.270001	-3.065358	-0.380746
O	-0.431675	0.663375	2.146915
O	2.612013	0.171952	0.109980
O	0.029640	-0.379194	-2.394917
O	-0.366994	2.525834	0.179882
O	1.012772	-1.863559	0.235165
O	-1.170386	-3.181881	0.100359
O	0.401266	-2.972475	-1.966746
O	1.070884	-4.423224	-0.016996
O	-1.941026	-0.805162	0.512016
H	-1.172090	0.046111	1.967738
H	0.089473	0.281804	2.860960
H	0.262120	-2.015650	-2.274615
H	-0.183666	-0.174823	-3.305513
H	-1.732375	-1.815413	0.390338
H	-2.822568	-0.666613	0.152536
H	2.468802	-0.800222	0.178152
H	3.378572	0.312953	-0.452493
H	-0.510171	3.424121	-0.129165
H	-0.506151	2.495812	1.138541
H	0.453941	-5.126562	0.213297

$CaH_3SiO_4(H_2O)_5^+$

Ca	0.182900	0.108637	0.002971
Si	0.260229	-3.286599	-0.584828
O	-0.286270	0.752023	2.315766
O	2.534955	0.024368	0.358788
O	-0.163233	-0.312835	-2.322725
O	-0.318270	2.491461	-0.154123
O	0.885580	-1.978968	0.065185
O	-1.283245	-3.417504	0.094634
O	0.042405	-3.028776	-2.234842
O	1.038328	-4.731719	-0.518458
O	-1.965783	-0.864262	0.667093
H	-1.035513	0.205321	2.591223
H	0.293977	0.842983	3.080413
H	-0.063746	-1.276784	-2.515119
H	-0.467007	0.110915	-3.130813
H	-1.878458	-1.845220	0.553634
H	-2.880514	-0.644849	0.460744
H	2.486563	-0.952850	0.293922
H	3.452098	0.308985	0.324341
H	-0.286217	3.172989	-0.835076
H	-0.550682	2.935322	0.671042
H	1.409682	-5.070652	0.299074

H	0.291034	-3.728722	-2.845756
H	-1.807888	-4.203433	-0.085968

$CaH_2SiO_4(H_2O)_5$

Ca	0.211027	0.128853	-0.101128
Si	0.335214	-3.208890	-0.486052
O	-0.712275	1.007387	2.158274
O	2.641991	0.118932	0.181007
O	-0.052031	-0.330696	-2.452320
O	-0.136685	2.542366	0.085568
O	1.136240	-1.897973	-0.048389
O	-1.122762	-3.345320	0.283128
O	0.097960	-3.011740	-2.162398
O	1.070592	-4.690663	-0.360236
O	-1.813702	-0.590877	0.629611
H	-1.386136	0.329157	1.748042
H	-0.529710	0.769191	3.070828
H	0.014659	-1.316247	-2.538530
H	-0.675727	-0.031240	-3.118909
H	-1.496913	-2.472766	0.533369
H	-2.736812	-0.537900	0.374237
H	2.483581	-0.859340	0.090687
H	3.526638	0.330196	-0.123399
H	0.086426	3.452398	-0.122029
H	-0.424159	2.486508	1.018372
H	0.858511	-5.190909	0.431172
H	-0.260697	-3.764333	-2.639954

$CaNO_3(H_2O)_4^+$

Ca	0.250541	-0.008681	0.094450
O	-0.258244	0.056475	2.431848
O	2.667369	-0.053957	0.021097
O	0.597770	2.385969	-0.114579
O	0.520870	-2.423801	0.097692
O	-0.467570	-0.039505	-2.111973
O	-1.982779	0.036498	0.475171
O	-4.153353	0.068698	0.363608
O	-2.991881	0.013780	-1.468271
N	-3.096436	0.040302	-0.215366
H	-1.231622	0.075897	2.388328
H	0.009315	0.093133	3.355735
H	-1.471936	-0.024105	-2.019566
H	-0.244825	-0.085992	-3.046400
H	0.308394	-2.958268	-0.678062
H	0.365278	-2.994649	0.860559
H	3.256088	0.705289	-0.068750
H	3.230402	-0.837477	0.035157

H	0.523707	3.046458	0.585405
H	0.340011	2.835343	-0.929757

$CaH(ox)(H_2O)_4^+$

Ca	0.018447	0.058836	0.267766
O	1.441349	1.406440	1.710807
O	1.246377	-1.238569	1.603292
O	2.809508	-1.442541	3.234348
O	2.979813	1.281085	3.331482
O	-0.351871	2.419672	-0.132581
O	-0.266503	-2.307546	-0.179016
O	-2.180089	0.009731	1.238704
O	0.997589	-0.122695	-1.912730
C	2.149240	0.743769	2.477211
C	2.101232	-0.829226	2.478754
H	-0.821823	3.047083	-0.692918
H	0.228983	2.928825	0.450338
H	1.194673	-1.010178	-2.238431
H	1.410103	0.496875	-2.525562
H	-0.721077	-3.081461	-0.525780
H	0.349299	-2.589033	0.526628
H	-2.757468	0.736438	1.501103
H	-2.573464	-0.794298	1.600569
H	2.950839	2.250297	3.266801

$CaH(ox)(H_2O)_5^+$

Ca	0.310339	-0.267907	-0.477089
O	0.653379	-0.150391	1.981699
O	0.613921	-0.805243	-2.814378
O	-1.551009	-1.552854	-1.420650
O	2.560811	0.692658	-0.679984
O	-0.428680	2.030144	-0.635140
O	1.314469	-2.347093	0.225928
O	-1.662759	0.029078	0.774375
O	-3.834008	-0.604955	1.022461
O	-3.687452	-2.118612	-1.142222
C	-2.604326	-1.478182	-0.796484
C	-2.740794	-0.592917	0.483277
H	1.373659	-2.442137	1.185180
H	1.359304	-3.231729	-0.154331
H	1.182611	0.254276	2.676696
H	-0.286414	-0.005738	2.196582

H	3.393674	0.209012	-0.729513
H	2.793941	1.622113	-0.572318
H	-0.459944	2.838883	-1.156608
H	-1.207474	2.019028	-0.056434
H	1.182129	-0.627428	-3.570758
H	-0.155481	-1.300584	-3.130054
H	-4.367056	-1.879506	-0.472881

$Ca(ox)(H_2O)_4$

Ca	-0.036112	-0.025682	0.188423
O	1.215145	1.324631	1.411893
O	1.236721	-1.289621	1.479748
O	3.007762	-1.332825	2.871794
O	2.607557	1.475666	3.176684
O	-0.758210	2.497477	0.401306
O	0.244189	-2.566114	-0.437208
O	-2.274247	0.360685	0.998061
O	0.771876	-0.472534	-2.039279
C	2.017094	0.836941	2.338301
C	2.147284	-0.743621	2.261946
H	-0.782170	3.347022	-0.048083
H	0.070055	2.459690	0.961253
H	0.863458	-1.437880	-1.945961
H	1.466715	-0.154800	-2.623439
H	-0.178484	-3.429520	-0.427597
H	0.809088	-2.481689	0.384118
H	-2.217998	1.331126	1.061933
H	-2.845722	0.040471	1.702241

$CaH(cit)(H_2O)_3$

Ca	1.428607	-0.535217	-0.255144
O	-0.435069	-0.446546	1.068751
O	-2.506803	-0.367200	1.929379
O	0.372738	1.043127	-1.459559
O	0.171117	2.744490	-0.034624
O	-1.933768	-3.054216	-0.674772
O	0.030466	-2.239721	-1.240012
O	-3.694010	0.111362	-0.289082
O	3.641709	-0.121176	-1.165943
O	1.726099	-1.587162	1.908106
O	2.432606	1.595885	0.547415
C	-2.280295	0.166212	-0.389722
C	-1.841757	1.625033	-0.717396
C	-1.873943	-0.793167	-1.572706
C	-1.700569	-0.255626	1.009384

C	-1.178234	-2.064103	-1.160941
C	-0.326153	1.845110	-0.742259
H	-2.283157	2.289873	0.024918
H	-2.261424	1.885170	-1.694782
H	-1.202730	-0.275063	-2.251492
H	-2.804075	-1.033083	-2.092595
H	3.677604	0.781409	-0.800211
H	4.101821	-0.120380	-2.010183
H	0.793568	-1.368605	2.127831
H	2.005973	-2.337136	2.438552
H	2.666250	1.805639	1.457542
H	1.660550	2.210207	0.299472
H	-3.860861	0.039391	0.671281
H	-2.858612	-2.773242	-0.617244

$Ca(cit)(H_2O)_3^-$

Ca	1.540776	-0.065020	0.419110
O	-0.441231	0.098112	1.397221
O	-2.628768	0.121667	1.897764
O	0.736850	1.335589	-1.221481
O	0.103078	3.095263	-0.009842
O	-1.316685	-2.956566	-0.154410
O	0.610842	-1.932518	-0.656078
O	-3.464670	0.218240	-0.521476
O	2.707046	-0.578393	-1.746325
O	1.889943	-2.229899	1.609387
O	2.144394	2.114679	1.360519
C	-2.044212	0.271823	-0.420607
C	-1.624877	1.696970	-0.922165
C	-1.446360	-0.861076	-1.336149
C	-1.684371	0.139464	1.103210
C	-0.700939	-2.023120	-0.653842
C	-0.164327	2.092469	-0.710440
H	-2.257217	2.423013	-0.411382
H	-1.854941	1.737708	-1.992500
H	-0.779407	-0.408129	-2.069025
H	-2.307932	-1.286828	-1.851218
H	2.278779	0.196114	-2.143118
H	2.013884	-1.269442	-1.788327
H	1.284347	-2.354015	2.348784
H	1.374071	-2.538102	0.815009
H	1.898877	2.307332	2.271017
H	1.489785	2.648273	0.808454
H	-3.749086	0.157660	0.411869

$Ca(cit)(H_2O)_4^-$

Ca	0.369042	0.389735	0.077333

O	0.027103	0.039369	2.547645
O	1.360567	-0.266260	-2.279434
O	-0.091125	1.908611	-1.570393
O	-0.220130	3.875165	-0.530628
O	-3.790905	1.221989	1.343009
O	-1.578641	1.303753	1.000939
O	-1.248142	-0.700174	-3.027495
O	-1.290401	-0.970756	-0.812018
O	-4.443846	2.097527	-0.980592
O	1.015466	-2.081203	-0.043441
O	1.570356	2.401127	0.801973
C	-0.747549	2.919421	-1.142342
C	-2.254002	2.916422	-1.386358
C	-3.070900	1.687644	-0.891608
C	-2.797200	1.369331	0.620511
C	-2.992742	0.412829	-1.801309
C	-1.743682	-0.469121	-1.908359
H	-2.682634	3.795663	-0.905456
H	-2.425416	3.009306	-2.464774
H	-3.266900	0.735558	-2.807130
H	-3.792962	-0.240183	-1.436902
H	1.600623	-2.055424	-0.811140
H	0.112910	-2.071831	-0.444060
H	1.478070	2.651324	1.726511
H	1.047972	3.084807	0.282830
H	-4.803287	1.846864	-0.108361
H	-0.822804	0.506988	2.328129
H	-0.212746	-0.826091	2.890800
H	1.332218	0.675401	-2.506447
H	0.509126	-0.588974	-2.673475

$CaH(mal)(H_2O)_4^+$

Ca	2.348300	0.878364	-1.985160
O	-0.513128	1.948141	0.973415
O	0.840437	0.632204	-0.128906
O	3.158283	2.275983	-0.148861
O	1.860042	-0.920017	-3.414949
O	4.453922	-0.145728	-1.383747

O	2.782379	2.563506	-3.614931
O	-0.659210	-0.206765	-3.673507
O	0.338287	1.588334	-2.768213
O	-3.000075	0.363098	-2.494283
C	-0.318065	0.967546	0.089766
C	-1.514698	0.367232	-0.595347
C	-1.851870	0.991763	-2.005467
C	-0.641354	0.785416	-2.923688
H	-1.322899	-0.696703	-0.744008
H	-2.414151	0.468327	0.015975
H	4.928842	0.176593	-0.607724
H	4.941985	-0.910932	-1.709397
H	2.155193	-1.770858	-3.751816
H	0.920971	-0.773256	-3.730434
H	1.891639	2.727449	-3.961728
H	3.414778	2.997305	-4.196684
H	2.524043	2.204281	0.578790
H	3.572742	3.143545	-0.078588
H	-2.051330	2.060421	-1.894299
H	-2.705420	-0.297494	-3.141617
H	-1.453140	2.129921	1.111724

$Ca(mal)(H_2O)_4$

Ca	2.505200	1.000984	-2.103105
O	0.355813	1.752599	0.614225
O	1.178193	0.015451	-0.525794
O	2.649106	2.611387	-0.343345
O	2.074030	-0.603399	-3.848769
O	3.760493	-0.562126	-0.715254
O	2.744541	2.705352	-3.838676
O	-0.552917	-0.084544	-3.743169
O	0.459022	1.712036	-2.871324
O	-2.814153	0.411442	-2.362936
C	0.199939	0.733112	-0.083682
C	-1.190789	0.322456	-0.547164
C	-1.614251	0.996636	-1.891957
C	-0.499554	0.867125	-2.938195
H	-1.214155	-0.757914	-0.699633
H	-1.933364	0.598652	0.202200
H	4.506826	-0.554460	-0.109523
H	2.942929	-0.630935	-0.178939
H	2.256720	-1.547074	-3.879410
H	1.093252	-0.494950	-4.007526
H	1.774581	2.653780	-3.953799
H	3.135509	2.818400	-4.709537
H	1.833276	2.366352	0.197417
H	2.686820	3.571635	-0.372344

H	-1.799127	2.057694	-1.714647
H	-2.544435	-0.249014	-3.018794
